# Genotoxicity and mutagenicity of *Echinodorus macrophyllus* (*chapéu-de-couro*) extracts

**DOI:** 10.1590/S1415-47572010005000060

**Published:** 2010-09-01

**Authors:** Leonardo S. Vidal, Adriana M. Alves, Ricardo M. Kuster, Claudia Lage, Alvaro C. Leitão

**Affiliations:** 1 Laboratório de Radiobiologia Molecular, Instituto de Biofísica Carlos Chagas Filho, Universidade Federal do Rio de Janeiro, Rio de Janeiro, RJBrazil; 2 Núcleo de Pesquisas de Produtos Naturais, Universidade Federal do Rio de Janeiro, Rio de Janeiro, RJBrazil

**Keywords:** Echinodorus macrophyllus, genotoxicity, mutagenesis, reactive oxygen species

## Abstract

*Echinodorus macrophyllus*, commonly known as *chapéu-de-couro*, is a medicinal plant used in folk medicine to treat inflammation and rheumatic diseases. In this work, we used short-term bacterial assays based on the induction of SOS functions to examine the genotoxicity and mutagenicity of an aqueous extract of *E. macrophyllus* leaves. Whole extract and an ethyl acetate fraction showed similar genotoxicity and caused an ~70-fold increase in lysogenic induction. The extract also gave a positive result in the SOS chromotest with an increase of 12-fold in β-Galactosidase enzymatic units. There was a strong trend towards base substitutions and frameshifts at purine sites in the mutations induced by the extract in *Escherichia coli* (CC103 and CC104 strains) and *Salmonella typhimurium* test strains (22-fold increase in histidine revertants in TA98 strain). Since reactive oxygen species may be implicated in aging process and in degenerative diseases, we used antioxidant compounds as catalase, thiourea and dipyridyl in the lysogenic induction test. All this compounds were able to reduce the induction factor observed in the treatment with *chapéu-de-couro*, thus suggesting that the genotoxicity and mutagenicity were attributable to the production of reactive oxygen species that targeted DNA purines.

## Introduction

In recent years, there has been a widespread increase in the use of medicinal plants or natural products because of their potentially beneficial effect on human health. However, there is little information on the mutagenic effects of most of the active principles found in these medicinal plants. Many plants may be effective phytomedicines but need to be exhaustively investigated to detect any toxic side effects. This is particularly important because many plants synthesize toxic substances for defense against viruses, bacteria and fungi and these compounds could have potentially deleterious effects in humans.

The biological effects of medicinal plant extracts are complex because of the presence of additional substances generated during processing and/or through the use of additives (Sugimura, 1982). Since many constituents found in industrial food/phytomedicine preparations may be mutagenic it is necessary to assess whether they can induce DNA damage in order to minimize the risks of cellular lesions during human consumption (Halliwell and Gutteridge, 1989). Many of these compounds have been associated with the generation of harmful reactive oxygen species (ROS) (Leitão and Braga, 1994; Fonseca *et al.*, 2000).

*Echinodorusmacrophyllus*, an aquatic plant of the family Alismataceae, known in Brazil as *chapéu-de-couro*, is widely used in the production of a very popular Brazilian soft drink. An aqueous extract of *E. macrophyllus* is used to treat rheumatic diseases that are usually characterized by exacerbated T and B lymphocyte responses. Pinto *et al.* (2007) observed immunosuppression of the T-cell response in mice treated orally with *chapéu-de-couro* extract for 7 days.

Since massive ROS production leading to DNA lesions is a major cause of toxic effects caused by plant extracts and products, in this work we used bacterial tests to evaluate the genotoxicity and mutagenicity of *chapéu-de-couro* extract. Genotoxicity was assessed using the inductest (Moreau *et al.*, 1976) and the SOS chromotest (Quillardet *et al.*, 1982), whereas mutagenicity was assessed by the reverse mutation test with histidine-auxotrophic strains of *Salmonella typhimurium*, *i.e.*, the Ames test (Maron and Ames, 1983), the WP2 mutagenicity test (Blanco *et al.*, 1998) and lactose mutagenesis assays (Cupples and Miller, 1989; Cupples *et al.*, 1990). The results obtained indicate that the extract has marked genotoxic and mutagenic effects that are clearly associated with structural alterations in purine targets.

## Material and Methods

###  Bacterial strains

The *Escherichia coli* strains used in this work are listed in Table 1 and the *S. typhimurium* strains are listed in Table 2.

###  Bacterial media

Bacteria were grown overnight in LB medium at 37 °C in a shaking incubator (Miller, 1992). After this incubation, an aliquot of this culture was inoculated into fresh LB medium (1:40 v/v) until the bacteria reached the exponential phase of growth.

The *E. coli* survival and infective center production assays were done by plating bacterial samples in LB medium and LB medium containing 10 μg of ampicillin/mL (LB-amp), respectively. Both media were solidified with 1.5% Difco bacto agar. We used E medium (Vogel and Bonner, 1956) solidified with 1.5% Difco bacto agar and supplemented with histidine for the Ames test (Maron and Ames, 1983) and with tryptophan for the WP2 mutagenicity test (Blanco *et al.*, 1998). A selective minimal medium containing 0.4% lactose was used for the Lac+-revertant mutagenesis assay (Cupples and Miller, 1989; Cupples *et al.*, 1990).

###  Preparation of *Echinodorus macrophyllus* infusion

Infusions of *E. macrophyllus* were prepared by the same standard procedure used by the pharmaceutical industry to obtain the phytomedicine for commercial use. Dried leaves of certified *E. macrophyllus* plants were kindly provided by a local pharmaceutical industry (Laboratório Simões Ltd., Rio de Janeiro, Brazil). A crude aqueous infusion was prepared by adding boiling water to grated dried leaves (200 mg/mL) for 10 min, after which the mixture was filtered, lyophilized and stored at -20 °C until further use.

###  Partition of the extract by solubility

The lyophilized extract was successively partitioned by an extraction method based on organic solvents of increasing polarity (hexane, chloroform, ethyl acetate and butanol). The extract was obtained by macerating leaves with 70% ethanol for 15 days, and the dry ethanolic extract was suspended in bidistilled water and subjected to successive partitions with the specified organic solvents. The samples obtained with each partitioning were subsequently screened for genotoxicity in the Inductest.

###  Chemicals

Catalase (Sigma Chemical Co., St. Louis, MO, USA) and thiourea (Indústrias Químicas Merck S/A, Rio de Janeiro, Brazil) were dissolved in bidistilled water. Dipyridyl (Sigma) was dissolved in 10% ethanol and then diluted in bidistilled water for each experiment.

###  Genotoxicity studies

####  Lysogenic induction assay 

Strains WP2s(λ) and RJF013 of *E. coli* B/r (Table 1) were used in the lysogenic induction assay in a protocol similar to the quantitative inductest developed by Moreau *et al.* (1976). Each assay was done in duplicate, with the results representing the average of at least three experiments. The level of induction was expressed as the induction factor, *i.e.*, the ratio of the number of infective centers after treatment divided by the number of spontaneous infective centers.

The positive controls in the genotoxicity and mutagenicity assays consisted of exposing the cultures to either a single UV-C dose (2 J/m^2^) or a single concentration (0.1 μg/plate) of 4-nitroquinolein-1-oxide (4-NQO).

####  SOS chromotest assay 

Strain PQ37 of *E. coli* K-12 (Table 1) was used in the SOS chromotest studies, according to the protocol developed by Quillardet and Hofnung (1985). The results represent the average of at least three experiments. β-Galactosidase activity (expressed in enzymatic units) was plotted against the absorbance at 420 nm after each treatment. Viability was determined based on the mean values of survival inactivation (data not shown).

####  Mechanism of action underlying the genotoxicity of the extract 

The mechanism of action involved in the genotoxicity was investigated in experiments in which the lysogenic induction was measured after exposure to a fixed amount of *chapéu-de-couro* extract (150 mg/plate) in the absence (control) or presence of antioxidant compounds. Since many plant extracts are capable of generating damaging ROS, the ability of the *chapéu-de-couro* extract to stimulate the production of these radical was examined by adding increasing concentrations of catalase, thiourea or dipyridyl to the incubations. Each assay was done in duplicate and the results represent the average of at least three experiments.

###  Mutagenicity studies

####  Reverse mutagenesis to histidine prototrophy (Ames test) 

This assay was done as described by Maron and Ames (1983), using the histidine *S. typhimurium* auxotroph mutant strains TA97, TA98, TA100 and the wild type strain TA102 (Table 2). Each assay was done in duplicate and the results represent the average of at least three experiments. Mutagenesis induced by *chapéu-de-couro* extract in each strain was expressed as the fold increase in the number of scored *his*^+^-induced revertants versus the spontaneous revertants.

####  Reverse mutagenesis to tryptophan prototrophy (WP2 test) 

This assay was done as described by Blanco *et al.* (1998), using the tryptophan auxotroph strains WP2s, IC203, IC204, IC206 and IC208 of *E. coli* (Table 1). Each assay was done in duplicate and the results represent the average of at least three independent experiments. Mutagenesis induced by *chapéu-de-couro* extract in each strain was expressed as the fold increase in the number of scored *trp*^+^-induced revertants versus the spontaneous revertants.

####  Lactose mutagenesis assay 

This assay was done as described by Cupples and Miller (1989) and Cupples *et al.* (1990) using different strains constructed to detect specific base pair substitutions and frameshift mutations in the *E. colilacZ* locus. Mutagenic activity was expressed as the number of revertants per 10^8^ cells. The scored spontaneous revertants were subtracted from the induced revertents to determine the effective mutagenesis frequency.

## Results

###  Genotoxicity induced by *chapéu-de-couro* extract

**Figure 1:**
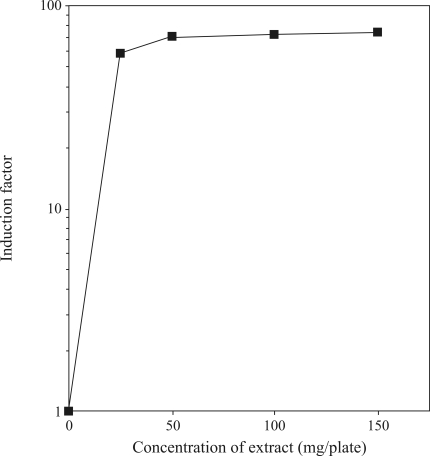
Dose-response curve for prophage induction as a function of the concentration of a lyophilized *chapéu-de-couro* extract. Cultures of *E. coli* WP2s(λ) in the exponential phase of growth were diluted (10^-4^) and 0.1 mL samples were pre-incubated at 37 °C for 20 min with the specified concentrations of lyophilized extract. The mixtures were then poured onto LB-amp plates together with 0.3 mL of indicator strain (RJF013) and 3 mL of molten soft agar.

**Figure 2:**
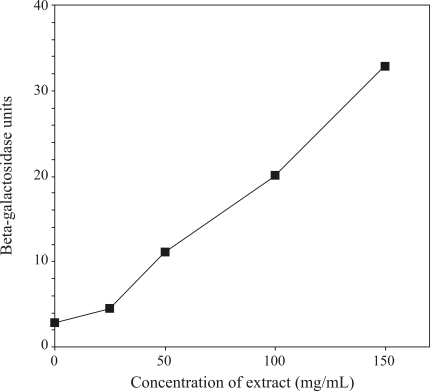
Induction of β-galactosidase in PQ37 strain after treatment with different concentrations of a lyophilized extract of *chapéu-de-couro*. The extent of β-galactosidase induction by the extract was calculated by using the equation developed by Quillardet and Hofnung (1985) after measuring the absorbance at 420 nm. Viability was assessed by survival inactivation (data not shown).

Figure 1 shows the prophage induction caused by different amounts of *chapéu-de-couro* extract. Maximal rates of prophage induction (corresponding to an ~70-fold increase above the spontaneous background) were observed with 50 mg of extract/plate. In the SOS chromotest, a concentration of 150 mg of lyophilized extract/mL increased the enzymatic activity to 33 units (a 12-fold increase above spontaneous enzymatic activity) (Figure 2). The results obtained with the Chromotest reinforced those of the inductest assay and indicated that *chapéu-de-couro* extract was genotoxic to bacterial cells.

###  Suppression of the genotoxicity of *chapéu-de-couro* extract

**Figure 3:**
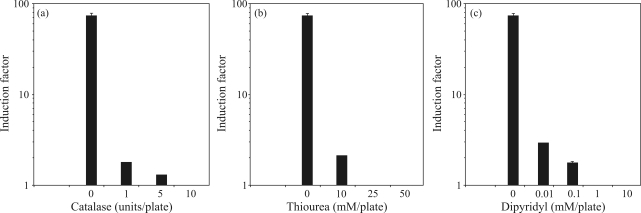
(a) Effect of catalase on the prophage-inducing activity of a lyophilized extract of *chapéu-de-couro*. Cultures of *E. coli* WP2s(λ) in the exponential phase of growth were diluted (10^-4^) and 0.1 mL samples were incubated with lyophilized extract (150 mg/plate) together with the indicated amounts of catalase for 20 min at 37 °C. The mixtures were then poured onto LB-amp plates together with 0.3 mL of indicator strain RJF013 and 3 mL of molten soft agar. The bars represent the standard errors. (b) Effect of thiourea on the prophage-inducing activity of a lyophilized extract of *chapéu-de-couro*. Cultures of *E. coli* WP2s(λ) in the exponential phase of growth were diluted (10^-4^) and 0.1 mL samples were incubated with lyophilized extract (150 mg/plate) together with the indicated amounts of thiourea for 20 min at 37 °C. The mixtures were then poured onto LB-amp plates together with 0.3 mL of indicator strain RJF013 and 3 mL of molten soft agar. The bars represent the standard errors. (c) Effect of dipyridyl on the prophage-inducing activity of a lyophilized extract of *chapéu-de-couro*. Cultures of *E. coli* WP2s(λ) in the exponential phase of growth were diluted (10^-4^) and 0.1 mL samples were incubated with lyophilized extract (150 mg/plate) together with the indicated amounts of dipyridyl for 20 min at 37 °C. The mixtures were then poured onto LB-amp plates together with 0.3 mL of indicator strain RJF013 and 3 mL of molten soft agar. The bars represent the standard errors.

To examine the mechanism by which *chapéu-de-couro* extract exerts its genotoxicity, different antioxidant agents were added to the bacterial cultures simultaneously with the extract (150 mg/plate). Figure 3 shows the inhibitory effects of catalase, dipyridyl and thiourea on the genotoxicity of the extract in the inductest assay. A single unit of catalase inhibited lysogenic induction by 50%, whereas nearly 100% inhibition was achieved with as little as 5 units of enzyme activity (Figure 3a). Thiourea and dipyridyl concentrations > 25 mM and > 1 mM, respectively, also completely blocked lysogenic induction (Figure 3b and 3c, respectively).

###  Genotoxicity of fractions of *chapéu-de-couro* extract

**Figure 4:**
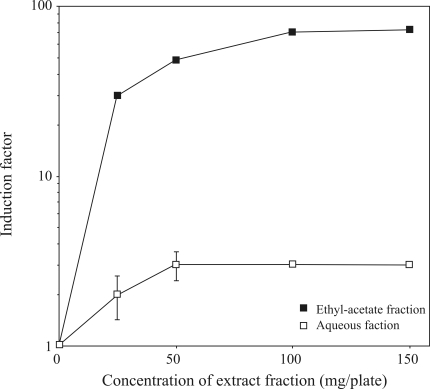
Dose-response curve for prophage induction by *chapéu-de-couro* extracts of different polarities*.* Cultures of *E. coli* WP2s(λ) in the exponential phase of growth were diluted (10^-4^) and 0.1 mL samples were incubated for 20 min at 37 °C with the indicated amounts of each fraction. The mixtures were then poured onto LB-amp plates together with 0.3 mL of indicator strain (RJF013) and 3 mL molten soft agar. The bars represent the standard errors.

To identify which fraction of *chapéu-de-couro* extract accounted for the genotoxicity of the whole mixture, inductest assays were done with fractions obtained by solubilizing the lyophilized extract in solvents of variable polarities. Figure 4 shows that the ethyl acetate fraction of *chapéu-de-couro* was more effective in inducing the prophage in *E. coli* WP2s(λ) when compared to the aqueous fraction (80-fold and 3-fold increases, respectively, at 150 mg/plate). The other fractions (hexane, chloroform and butanol) did not induce the prophage (data not shown). This ethyl acetate fraction is expected to contain aglycone flavonoids such as quercetin and lutheolin, and phenylpropanoids (Zhang *et al.*, 2001).

###  Mutagenicity assessed by the Ames test

**Figure 5:**
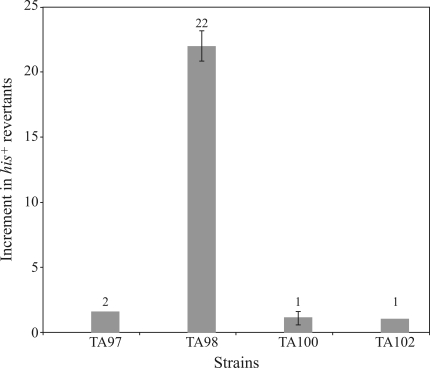
Mutagenic activity of a lyophilized extract of *chapéu-de-couro*. Cultures of *S. typhimurium* in the stationary phase of growth (0.1 mL samples) were pre-incubated for 20 min at 37 °C with 150 mg of lyophilized extract/plate. After pre-incubation, the mixtures were poured onto E medium plates together with 2 mL of molten top agar supplemented with *his*-bio solution (200 μL). Viability was assessed by survival inactivation (data not shown).

The mutagenicity of *chapéu-de-couro* extract assessed by the Ames test is shown in Figure 5. A 22-fold increase in the number of spontaneous revertants was observed after treatment of *E. coli* strain TA98 with lyophilized extract (150 mg/plate). This finding suggested that GC sites are targeted by components present in the extract. The extract was not mutagenic in strains TA97, TA100 and TA102. At the concentrations tested, the extract was not lethal to any of the strains examined (data not shown).

###  Mutagenicity in WP2s Trp^-^ strains

**Figure 6:**
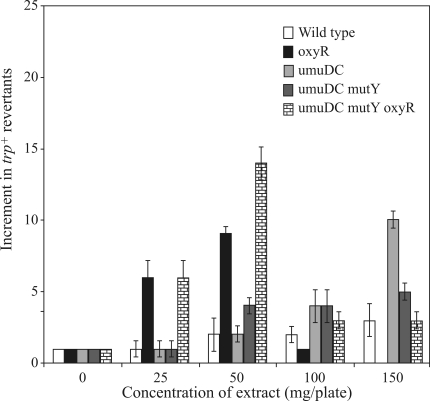
Mutagenic activity of a lyophilized extract of *chapéu-de-couro*. Cultures of *E. coli* in the stationary phase of growth (0.1 mL samples) were pre-incubated for 20 min at 37 °C with the indicated concentration of lyophilized extract. After pre-incubation, the mixtures were poured onto E medium plates containing tryptophan (0.5 mg/L) and 2.5 mL of molten top agar.

The lyophilized extract of *chapéu-de-couro* did not increase the number of revertants in the wild-type WP2s strain at any of the concentrations tested. A deficiency in OxyR protein function caused the number of revertants in strain IC203 to increase 6- and 10-fold, relative to the spontaneous rate after treatment with 25 and 50 mg of extract/plate, respectively. Since the *oxy*R mutation renders this strain more sensitive to oxidative stress caused by H_2_O_2_ or other organic peroxides these findings suggest that some of the mutagenicity may be mediated by peroxide activity. Similar amounts of extract (25 and 50 mg/plate) also increased the number of revertants in strain IC208 by 6- and 13-fold, respectively. This induction probably resulted from a deficiency in UmuDC and MutY functions. Remarkably, at the highest amount of extract tested (150 mg/plate) there was a 10-fold increase in the number of revertants in *umu*DC-deficient strain IC204, which suggested that SOS-independent pathways may also be involved in this response. The extract did not significantly affect strain IC206, although there was a discrete 5-fold increase in mutagenesis after treatment with > 50 mg of extract/plate (Figure 6); as with strain IC204, this finding may indicate the involvement of SOS-independent mechanisms in this damage.

###  Lactose mutagenesis assay: definition of mutagenic hot spots

**Figure 7:**
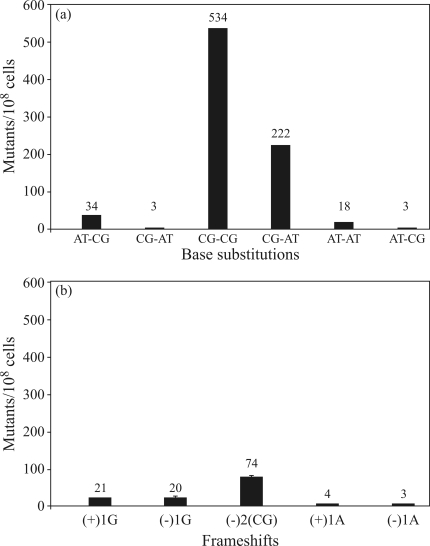
Mutagenic activity of a lyophilized extract of *chapéu-de-couro*. Cultures of *E. coli* in the exponential phase of growth were pre-incubated for 20 min at 37 °C with 150 mg of lyophilized extract/plate. After pre-incubation, aliquots of 200 μL were added to 2 mL of LB medium and incubated overnight prior to pouring onto minimal medium plates containing 0.4% lactose. (a) base substitutions, (b) frameshifts.

To investigate which DNA bases were targets for components of the *chapéu-de-couro* extracts, the lactose mutagenesis assay was done using *E. coli* strains with specific Lac^-^ → Lac^+^ reversion phenotypes (Figure 7a,b). As shown in Figure 7a, the *chapéu-de-couro* extract specifically reverted strains CC103 (534 revertants) and CC104 (222 revertants) to the Lac^+^ phenotype.

## Discussion

The results of this study indicate that *chapéu-de-couro* (*E. macrophyllus*) extract is genotoxic and mutagenic to *E. coli*. The extract induced λ prophage in the *E. coli* strain WP2s(λ) and induced the expression of β-galactosidase in *E. coli* strain PQ37. The treatment of *S. typhimurium* strains CC103 and CC104 with *chapéu-de-couro* extract indicated that GC sites may be the targets for mutagenic compounds since these strains are sensitive to base substitution at purine sites.

ROS are deleterious to many organisms and have been implicated in aging and in degenerative diseases such as cancer (Harman, 1956; Valko *et al.*, 2007). The consecutive univalent reduction of molecular oxygen to water produces three active intermediates: superoxide anion (O_2_^•–^), hydrogen peroxide (H_2_O_2_) and hydroxyl radical (OH^•^). These oxygen species are potent oxidants of lipids, proteins and nucleic acids, and may be related to the genotoxicity of several substances present in human foods (Ames and Gold, 1991).

Previous studies have shown that catalase reduces the genotoxicity of tea, *mate*, *guaraná* and coffee infusions, which suggests the participation of ROS in the toxicity of these extracts (Fonseca *et al.*, 1994; Leitão and Braga, 1994; Fonseca *et al.*, 2000). Our results indicate that ROS may also be involved in the genotoxicity of *chapéu-de-couro* extract since lysogenic induction was effectively inhibited by catalase (10 U/plate). This finding suggests that hydrogen peroxide present in, or generated by, compounds in the extract may have a role in the resulting genotoxicity.

The genotoxicity and mutagenicity of coffee may be attributable to the presence of hydrogen peroxide and the hydroxyl radical (OH^•^), as suggested by the use of free radical scavengers (Fujita *et al.*, 1985; Ariza *et al.*, 1988; Ruiz-Laguna and Pueyo, 1999). As shown here, thiourea suppressed the genotoxicity of *chapéu-de-couro* extract, indicating a possible role for OH^•^ in this effect. This conclusion agrees with the previously reported ability of pretreatment with thiourea to protect *E. coli* against the lethal effects of H_2_O_2_ (Brandi *et al.*, 1989). OH^•^ can be generated by the oxidation of transition metals that act as reducing agents for H_2_O_2_ in the Fenton reaction (Imlay *et al.*, 1988). The fact that dipyridyl reduces OH^•^ production via the Fenton reaction strongly supports the hypothesis that *chapéu-de-couro* extract may exert its genotoxicity through ROS formation.

Some compounds previously recognized as carcinogens in eukaryotic assays have tested negative when screened in bacterial mutagenic assays, even when genotoxic in the inductest, *e.g.*, several metallic substances (Rossman *et al.*, 1984). A previous study found no mutagenicity for *chapéu-de-couro* extract (up to 50 mg/plate) in *S. typhimurium* TA strains (da Costa Lopes *et al.*, 2000). As shown here with the Ames test, the *chapéu-de-couro* extract (150 mg/plate) was mutagenic only in strain TA98, which indicates that GC sites are the main targets in DNA. According to Gatehouse *et al.* (1994), the ability of a substance to double the number of revertants compared to the spontaneous rate is the most widely accepted criterion for considering a substance to be mutagenic in the Ames test. Based on this definition, we conclude that the *chapéu-de-couro* extract is mutagenic.

To confirm the results obtained with the Ames test, experiments were done using tryptophan auxotrophic *E. coli* WP2s strains with different DNA repair backgrounds.

One of the main oxidative damages induced by endogenous and exogenous compounds is the formation of 8-oxoguanine (8-oxo-dG). This damage is unrelated to cellular lethality in *E. coli*. Rather, this alteration is highly mutagenic, especially in the absence of MutT, MutM (Fpg) or MutY proteins. The importance of these proteins in minimizing such oxidative damage is attested to by their absolute evolutionary conservation (Tchou and Grollman, 1993; Blanco *et al.*, 1998). The mutagenic activity of the *chapéu-de-couro* extract apparently targeted GC sites since the Ames assay was positive in TA98, and reversion to tryptophan prototrophy was greater in strains harboring defective anti-oxidative responses. In the case of the *mut*Y strain, the specific increase in G:C → T:A transversions suggested the accumulation of 8-oxoguanine and of 8-oxo-dG:A mispairings.

To examine the DNA-specific targets further, a group of *E. coli* strains carrying specific point mutations in the lactose operon were tested against the *chapéu-de-couro* extract. Again, the results indicated that compounds in the *chapéu-de-couro* extract were able to induce transversions at GC sites, especially G:C → C:G and G:C → T:A mutations, as well as GC deletions (Figure 7a,b). In many respects, the spectrum of mutations generated by the extract resembled that induced by ROS attack of guanine targets (McBride *et al.*, 1991; Retel *et al.*, 1993; Akasaka and Yamamoto, 1994). The latter noted that the increase in mutation frequency after treatment with H_2_O_2_ correlated closely with the increase in 8-oxoguanine formation.

McBride *et al.* (1991) observed G-to-C transversions followed by C-to-T transitions in an analysis of ROS-induced mutations in the *lac*Z gene, with G-to-T transversions being the most prevalent mutational events. Analysis of sequences from 82 mutants showed base substitutions to be the most prominent mutational event in 70 cases, with 63 of these being G:C transversions. The G:C-to-C:G transversion was the most frequent (28 cases), followed by 26 cases of G:C-to-T:A. These authors suggested that G:C-to-T:A transversions were a consequence of mispairing between a modified guanine, probably 8-oxo-guanine, and deoxyadenosine. In contrast, the origin of G:C-to-C:G transversions was attributed to the formation of unidentified damage generated by H_2_O_2_.

In addition to the mechanisms indicated above, the components of the *chapéu-de-couro* extract may also directly generate abasic sites in DNA, particularly through guanine residues. This could explain the G-to-C transversions observed after treatment with *chapéu-de-couro* extract. These transversions were also observed by Murry (1986) after treatment with hydroxylaminopurine (HAP), a compound with anti-tumoral activity in rat lungs. However, we cannot eliminate the possibility that other types of damage, as yet uncharacterized in terms of their mutagenic potential, can give rise to G:C-to-C:G transversions such as observed here.

As shown in Figure 4, the ethyl acetate fraction of *chapéu-de-couro* extract was strongly genotoxic. Several studies have shown that quercetin causes base-pair substitutions and frameshift mutations in Ames strains (Bjeldanes and Chang, 1977; Hatcher and Bryan, 1985; Makena *et al.*, 2009), chromosomal aberrations and sister chromatid exchanges in CHO cells (Carver *et al.*, 1983), and micronucleus formation in human lymphocytes in the absence or presence of metabolic activation (Caria *et al.*, 1995). While the redox potentials of most flavonoid radicals are below those of superoxide and alkyl peroxide radicals (Jørgensen *et al.*, 1998), their effectiveness in generating lipid peroxidation, DNA adducts, and mutations may be biologically relevant. Quercetin, an aglycone form of a flavonoid glycoside, was the most mutagenic compound to TA98 *S. typhimurium* strain (Hatcher and Bryan, 1985); this strain was also sensitive to the *chapéu-de-couro* extract. Quercetin is also mutagenic in test strains of *E. coli* (Makena *et al.*, 2009). Particular attention should therefore be paid to quercetin since it is the most predominant aglycone flavonoid in the human diet, with an estimated human consumption of 4-68 mg/day based on epidemiological studies in the US (Hertog *et al.*, 1993, 1995; Rimm *et al.*, 1996; Knekt *et al.*, 1997). Other compounds such as diterpenoids have also been isolated from *E. macrophyllus* (Kobayashi *et al.*, 2000; Shigemori *et al.*, 2002). A diterpenoid isolated from *Sagittaria pygmaea* showed antibacterial acitivity against oral pathogens, but no biological activities have been attributed to diterpenoids from *E. macrophyllus*. In the case of *chapéu-de-couro* extract, it is one of the main ingredients used to prepare a very popular soft drink (*Mineirinho*) widely consumed in Brazil.

In conclusion, our results indicate that *chapéu-de-couro* extract is genotoxic and mutagenic in bacterial tests. The aglycones lutheolin and quercetin may be responsible for this activity and could be potentially carcinogenic in uncontrolled human consumption. Further studies are needed to evaluate the carcinogenicity of this extract in order to adequately assess the risks for human health.

## Figures and Tables

**Table 1:** *Escherichia coli* strains used in this work.

Designations	Relevant genotype	Reference
WP2s	WP2 *uvr*A / pKM101 *amp*^R^	Blanco *et al.* (1998)
IC203	WP2 *oxy*R / pKM101 *amp*^R^	Blanco *et al.* (1998)
IC204	WP2 Δ (*umu*DC) *cat*^R^	Blanco *et al.* (1998)
IC206	WP2 Δ (*umu*DC) *mut*Y *cat*^R^	Blanco *et al.* (1998)
IC208	WP2 Δ (*umu*DC) *mut*Y *oxy*R *cat*^R^	Blanco *et al.* (1998)
CC101	P90C *ara* Δ (*lac pro*B)_XIII_ (A: T → C:G)	Cupples and Miller (1989)
CC102	P90C *ara* Δ (*lac pro*B)_XIII_ (G:C → A:T)	Cupples and Miller (1989)
CC103	P90C *ara* Δ (*lac pro*B)_XIII_ (G:C → C:G)	Cupples and Miller (1989)
CC104	P90C *ara* Δ (*lac pro*B)_XIII_ (G:C → T:A)	Cupples and Miller (1989)
CC105	P90C *ara* Δ (*lac pro*B)_XIII_ (A:T → T:A)	Cupples and Miller (1989)
CC106	P90C *ara* Δ (*lac pro*B)_XIII_ (A:T → G:C)	Cupples and Miller (1989)
CC107	P90C *ara* Δ (*lac pro*B)_XIII_ (+ 1G)	Cupples *et al.* (1990)
CC108	P90C *ara* Δ (*lac pro*B)_XIII_ (-1G)	Cupples *et al.* (1990)
CC109	P90C *ara* Δ (*lac pro*B)_XIII_ (-2(CG))	Cupples *et al.* (1990)
CC110	P90C *ara* Δ (*lac pro*B)_XIII_ (+1A)	Cupples *et al.* (1990)
CC111	P90C *ara* Δ (*lac pro*B)_XIII_ (-1A)	Cupples *et al.* (1990)
WP2s(λ)	WP2 *uvr*A (λ) *trp*E	Our laboratory stock
RJF013	[B/r SR 714] *uvr*D3 *trp*E *amp*^R^	Our laboratory stock
PQ37	*uvr*A *rfa sfi*A*::lac*Z	Quillardet and Hofnung (1985)

**Table 2:** *Salmonella typhimurium* strains used in this work.

Designations	Relevant genotype	Reference
TA97	*his*D6610/ *his*O1242 - Δ*uvr*B *rfa* pKM101 *(amp*^*R*^)	Maron and Ames (1983)
TA98	*his*D3052 - Δ*uvr*B *rfa* pKM101(*amp*^R^)	Maron and Ames (1983)
TA100	*his*G46 - Δ*uvr*B *rfa* pKM101 (*amp*^R^)	Maron and Ames (1983)
TA102	*his*G428-wild type *rfa* pKM101(*amp*^R^) pAQ1 (*tet*^R^)	Maron and Ames (1983)
